# Comparison of social and sociodemographic characteristics and treatment goals of persons with alcohol versus drug use disorders: Result from a national census of inpatients in specialized treatment for substance use

**DOI:** 10.1016/j.abrep.2021.100340

**Published:** 2021-02-03

**Authors:** Helle Wessel Andersson, Solfrid E. Lilleeng, Solveig Osborg Ose

**Affiliations:** a Department of Research and Development, Clinic of Substance Use and Addiction Medicine, St. Olavs University Hospital, PB 3250 Sluppen, 7006 Trondheim, Norway; b The Norwegian Directorate of Health, Department of Analysis and Performance Assessment, Holtermanns vei 70, Trondheim 7031, Norway; c SINTEF Technology and Society, Department of Health, Klæbuveien 153, Trondheim 7049, Norway

**Keywords:** Alcohol, Illicit drugs, Inpatient treatment, Social relationships, Sociodemographic characteristics, Treatment goal

## Abstract

**Introduction:**

We examined differences in social and sociodemographic characteristics and treatment goals between people with primary alcohol use disorder (AUD) versus those with a primary drug use disorder receiving inpatient treatment for a substance use disorder (SUD).

**Methods:**

A national census utilizing a cross sectional design included 56 of 60 specialized inpatient SUD treatment clinics in Norway and all patients receiving treatment on a specific date (responserate = 70%*).* Data on substance use, social and sociodemographic characteristics, and patient-reported treatment goals were collected. Patients were classified as having primary AUD or a drug use disorder based on the main SUD diagnosis relevant to the treatment episode.

**Results:**

The analytic sample included 1093 patients. Patients with primary AUD (n = 362) were more often older, had a higher educational level and income from work, and lived in permanent housing compared with patients with a drug use disorder (n = 731). Patients with AUD were more likely to have good relationships with friends. The higher frequency of reported reduced substance use (versus quitting substance use) as the treatment goal among AUD patients disappeared when controlled for sociodemographic factors.

**Conclusions:**

Knowledge about the different characteristics of inpatients with AUD versus a drug use disorder is relevant when conducting research involving the SUD treatment population and for facilitating treatment. The lower frequency of perceived support from friends among patients with a drug use disorder suggests a need for targeted efforts in (re)building supportive social relationships for inpatients being treated for SUD.

## Highlights

• A national census of patients in residential substance use treatment.
• We compared inpatients with primary alcohol use disorders vs. drug use disorders.
• Patients with drug use disorders were more likely sociodemographic disadvantaged.
• Patients with drug use disorders more likely had poor friend relationships.
• Treatment goal differences disappeared when adjusted for sociodemographic variables.

## 1 Introduction

People admitted to specialized inpatient substance use disorder (SUD) treatment have substance use dependency of high severity and complexity (Reif et al., 2014, WHO, 2020). Most specialized SUD treatment units target both individual patients with a primary alcohol use disorder (AUD) and those with a primary illicit drug use disorder (de Andrade et al., 2019, Reif et al., 2014). In Norway, patients with primary AUD typically constitute 35–40% of the total patient population in specialized SUD treatment units (Norwegian Directorate of Health, 2017).

Long-term follow-up studies have documented the different trajectories of people with AUD versus a drug use disorder (Hser et al., 2001, Vaillant, 2003). Moreover, research has suggested that people with primary AUD have distinct patient characteristics compared with people with drug use disorders, and that they represent a less marginalized group in terms of sociodemographic characteristics (Swendsen et al., 2009). For example, one study based on a large sample representative of the US population reported that compared with those with primary AUD, people with a lifetime diagnostic status of illicit drug use disorder were younger and more likely to have a lower socioeconomic status (e.g. no college education, currently unemployed) and to be homeless (Simpson, Rise, Browne, Lehavot, & Kaysen, 2019). However, when it comes to the total harmful effects of a given substance to the individual user and others, studies have suggested that alcohol is causing the greatest overall harm (Bonomo et al., 2019, Nutt et al., 2010, Van Amsterdam et al., 2010).

A person’s physical resources, such as having money and property (Best & Laudet, 2010), and social capital, such as social support from friends and family (Brooks, Lòpez, Ranucci, Krumlauf, & Wallen, 2017) may be important factors affecting recovery from SUD. Thus, insight into differences in these areas between patients with AUD versus a drug use disorder may be relevant to the SUD treatment context. Differences in characteristics between people with an AUD versus a drug use disorder found in population-based studies may not generalize to the SUD inpatient population because of the possible bias relating to the selection of people who are referred to and can access specialized treatment (Maust et al., 2011, Van Boekel et al., 2013). However, only few studies have investigated the social and sociodemographic differences between people with a primary AUD and drug use disorder receiving SUD treatment. Consistent with research involving nonpatient samples, a study of diverse patients receiving SUD treatment in Australia showed that those seeking treatment for primary AUD had a higher educational level and employment status, and tended to report a better living situation, compared with those with a primary drug use disorder (Lubman et al., 2016). Among the available research on differences in social capital between these groups, one study has suggested that people who have drug use disorders appear to be more disadvantaged with regard to social relationships with friends than are those with AUD (Stenius, Witbrodt, Engdahl, & Weisner, 2010).

Given the possible differences in social and sociodemographic characteristics between people with primary AUD versus those with drug use disorders, the treatment goals may also differ. Generally, planning for inpatient SUD treatment includes formulation of individual treatment goals that represent realistic and acceptable changes in areas that are important for recovery and adaptation within the community. A reduction in or cessation of alcohol or drug use should be the main goal of specialized SUD treatment (Norwegian Directorate of Health, 2016, WHO, 2020). Among the limited research available on these issues, one study based on clinicians’ ratings suggested that reduced use was a more widely accepted treatment goal for patients with alcohol and cannabis addiction than for users of other illicit substances (Rosenberg & Davis, 2014). Increased knowledge about differences in treatment goals between patients with AUD versus a drug use disorder is relevant in both the clinical and research contexts.

While all different types of substance use disorders have distinct characteristics (WHO, 1992), research has suggested the therapeutic relevance of considering AUD and drug use disorders as two main classes of SUD disorders (Ozburn, Janowsky, & Crabbe, 2015). However, only a few treatment sample studies have investigated the differences between these groups, and the studies have included a limited number and types of variables. This study will provide further insight into the relative importance of social and sociodemographic factors in discriminating between inpatients with AUD and those with a drug use disorder, and extend current literature on differences in the treatment outcome goals between these groups.

The aim of this study was to compare the social and sociodemographic characteristics and treatment goals of patients with AUD versus a drug use disorder receiving inpatient SUD treatment. Given the previous findings, we hypothesized that, compared with patients with a primary drug use disorder, those with primary AUD would be characterized by being older and less disadvantaged as concerns sociodemographic factors (including education level and source of income), (Lubman et al., 2016) and social life (i.e. relationships with family and friends) (Stenius et al., 2010). We also hypothesized that, relative to persons with drug use disorders, those with AUD would more frequently report reduced substance use (as opposed to stop using substances) as a treatment goal (Rosenberg & Davis, 2014).

## 2 Material and methods

### 2.1 Study design

All patients receiving inpatient SUD treatment were the target group for a national census commissioned and financed by the Norwegian Directorate of Health and carried out by the SINTEF Research Foundation. The study had a cross-sectional design and included all patients undergoing treatment on a specific date (November 1, 2012). Data were collected anonymously after taking an informed consent from the patients. The Regional Committee for Medical and Health Research Ethics (reg. no. 2012/848) approved the current study.

### 2.2 Setting

In Norway, specialized SUD treatment falls under four regional health authorities (RHAs), which are responsible for the provision of health services for the people living in that area. The services are mostly public but also involve private trusts, which have an operating agreement with the RHA (Lindahl, 2016). Most of the treatment services cover both patients with primary AUD and those with drug use disorders, including patients dependent on prescribed drugs. The specialized SUD treatment comprises outpatient treatment, short-term treatment (up to 6 months), and long-term treatment (longer than 6 months). Norwegian reports state that annually, about 30,000 people receive specialized SUD treatment (1% of the adult population) (Statistics Norway, 2012), and that this figure has remained stable over the past 8 years (Norwegian Directorate of Health, 2017, Norwegian Directorate of Health, 2018). About 90% of those in need of specialized SUD treatment are provided outpatient treatment, but the bulk of the resources in specialized SUD treatment are consumed by inpatient treatment (Kalseth, Ose, Kalseth, Paulsen, & Magnussen, 2013).

Referrals of patients to specialized SUD treatment are made by social services, general practitioners, or specialized health services (Ministry of Health Care Services, 2004). An interdisciplinary assessment unit comprising staff with social, psychological, and medical expertise assess all referred clients. Planning for inpatient SUD treatment includes formulation of the individual treatment goals that represent realistic and acceptable changes in areas that are important for recovery of the patient’s life and adaptation into the community.

A reduction or cessation of alcohol or drug use should be the main goal of specialized SUD treatment based on an individual assessment of treatment needs (WHO, 2020) and specified in collaboration between the patient and the therapist (Norwegian Directorate of Health, 2016). As in other European countries, the SUD inpatient treatment programs in Norway provide comprehensive treatment and recovery programs that focus on individually based social, biological, and mental health needs through a combination of group and individual therapies (EMCDDA, 2014).

### 2.3 Data collection

All SUD treatment clinics providing inpatient treatment in the public and private sectors across the country were invited to participate in the census. Several months before the data collection, the service managers and clinicians received information describing the project and data collection procedures. The patient’s clinician was responsible for completing an anonymous registration form for each patient undergoing treatment on the actual date. The form included information on primary SUD of concern for the current treatment episode, sociodemographic characteristics, social relationships, and the patient’s treatment goals. The clinicians’ reporting was based on information from the medical record supplemented with information provided by the patient as part of this study.

### 2.4 Variables

#### 2.4.1 Primary SUD of concern

Information on the primary SUD of concern was based on the recorded SUD diagnosis (F10–F19) according to the International Classification of Diseases (10th revision, ICD-10) (WHO, 1992). In cases where a SUD diagnosis was missing (17%), each patient’s report of the most frequently used drug in the 4 weeks before admission was used. The primary SUD of concern was categorized according to the following categories of ICD-10: alcohol (F10); opioids (F11); cannabis (F12); sedatives (F13, benzodiazepines and other addictive drugs); stimulants (F14 and F15, cocaine, amphetamines and other stimulants); or multiple substance use (F19).

#### 2.4.2 Sociodemographic characteristics

Sociodemographic characteristics included in the analysis were gender, age, educational attainment, source of income, and marital status. Educational attainment was categorized as low educational level (only primary school), medium educational level (secondary school), and higher educational level (university or higher education). Income was categorized as income from labor, health-related benefits, and other economic support. Marital status was grouped into three categories: married/cohabitating/partnered, separated/divorced/widowed, single/unmarried. The patient’s housing situation was classified as living in owned home, living in rented housing in the private market, living in rented municipal housing, and without permanent residence.

#### 2.4.3 Social relationships

Measures of social relationships included the patient’s responses to the following two questions: “How is your relationship with your family?” and “How is your relationship with your friends?”. These questions were answered on a 4-point scale ranging from “Very good”, “Quite good”; “Quite bad”, to “Very bad”.

#### 2.4.4 Treatment goals

The treatment goal options included the two main goals: to quit using substances or to reduce substance use. A list of additional treatment goal options, more than one of which could be stated, included the following: increasing the quality of life; reducing the somatic symptoms; keeping custody of children; and other. Answers in the category “other” were coded and sorted into the abovementioned main categories.

### 2.5 Statistical methods

Patients were classified as having a primary AUD or primary drug use disorder based on information about the main SUD diagnosis/type of substance use of relevance for the current treatment episode. The chi-square test was used to compare social and sociodemographic characteristics, and treatment goals between groups. To assess how well each of the variables differentiated between the groups when controlling for the remaining independent variables, variables that differed significantly between the two groups (*p* < 0.10) in the bivariate analyses were examined further in multivariate logistic regression analysis. Analyses were performed using STATA (Stata/SE 14.2 for Windows; StataCorp LP, College Station, TX).

## 3 Results

### 3.1 Participants

The sample included 1139 patients from 56 of the 60 specialized inpatient substance use treatment departments in Norway. Based on data from the National Patient Register on the number of patients attending SUD inpatient treatment at the time of the census, the responserate was estimated to be 70% (Ose & Pettersen, 2014). Because of missing data about the primary substance of concern, 46 patients were excluded from further analyses. Table 1 shows the number of patients in the analytic sample (n = 1093) within the different SUD diagnostic categories.

**Table 1:** Primary substances of concern of relevance to the index treatment.

Substance use disorder	Number of patients	%
Alcohol (F10)	362	33
Opioids^1^ (F11)	227	21
Cannabis (F12)	118	11
Sedatives (F13)	64	6
Stimulants^2^ (F15)	162	15
Multiple substance use^3^ (F19)	160	14

*Total*	1093	100

*Note.*^1^ Includes misuse of methadone. 99 patients registered as currently on a clinically supervised or replacement regime (F11.22). ^2^ Includes F14 cocaine (n = 1). ^3^ Includes reported use of multiple substances.

About one third of the patients (n = 362) had alcohol as their primary SUD of concern. Among patients with primary drug use disorders (n = 731), opioids represented the most prevalent drug, followed by stimulants and cannabis. The use of two or more drugs (polysubstance use) was registered as the primary drug of concern for 160 patients.

### 3.2 Bivariate comparisons between patients with AUD versus a drug use disorder

Table 2 shows the comparisons of the social and sociodemographic characteristics between patients with AUD versus those with a drug use disorder.

**Table 2:** Chi-square comparison of social and sociodemographic characteristics of patients with primary alcohol use disorder versus primary drug use disorder.

Variable	Alcohol use disorder (n = 362)	Drug use disorder (n = 731)		
	N (%)	N (%)	χ^2^	*p* value
*Gender*			1.24	0.265
Female	106 (30)	191 (26)		
Male	252 (70)	533 (74)		

*Age*			324.1	<0.0001
18–23 years	17 (5)	155 (22)		
24–29 years	21 (6)	145 (20)		
30–39 years	52 (15)	251 (35)		
40–49 years	124 (35)	127 (18)		
≥50 years	142 (40)	34 (5)		

*Educational level*			98.69	<0.0001
High	61 (18)	20 (3)		
Medium	165 (47)	261 (37)		
Low	122 (35)	430 (60)		

*Main source of income*			47.58	<0.0001
Income from labor	46 (13)	35 (5)		
Health-related benefits	268 (74)	482 (67)		
Other economic support	46 (13)	202 (28)		

*Marital status*			83.18	<0.0001
Married/cohabitating/partnered	75 (21)	89 (12)		
Separated/divorced/widowed	102 (29)	74 (10)		
Single/unmarried	180 (50)	557 (77)		

*Housing situation*			133.48	<0.0001
Owned home	123 (34)	74 (10)		
Rented housing in private market	127 (35)	221 (31)		
Rented municipal housing	56 (16)	128 (18)		
Without permanent residence	52 (15)	298 (41)		

*Relationship with family*			5.05	0.169
Very good	118 (34)	193 (28)		
Good	158 (46)	349 (51)		
Poor	50 (15)	95 (14)		
Very poor	18 (5)	47 (7)		

*Relationship with friends*			26.43	<0.0001
Very good	59 (18)	69 (10)		
Good	159 (47)	260 (39)		
Poor	77 (23)	195 (29)		
Very poor	40 (12)	139 (21)		

The chi-square analyses showed that the two groups differed on most of the variables being investigated. Compared with patients with a drug use disorder, patients with AUD were more often older and had a higher educational level (medium/high) and higher income from work. Patients with AUD were less often single, more often living in an owned or rented apartment in the private market, and more frequently reported good relationships with friends compared with those with a drug use disorder.

Table 3 presents the results of the bivariate analysis comparing the reported treatment goals for patients with AUD versus those with a drug use disorder.

**Table 3:** Chi-square comparison of treatment goals of patients with primary alcohol use disorder versus drug use disorder.

Patient-reported treatment goals	Total sample (n = 1093)	Alcohol use disorders (n = 362)	Drug use disorders (n = 731)		
	n	%	%	χ^2^	*p* value
*Main treatment goal*					
Quit using substances	907	79.8	84.5	3.81	0.051
Reduce substance use	169	18.5	13.9		

*Additional treatment goals*^1^					
Increase quality of life	768	68.8	71.0	0.53	0.466
Reduce somatic symptoms^2^	365	30.9	34.6	1.45	0.229
Keep custody of children	115	6.1	12.7	12.33	<0.000

Note. 17 patients did not report their main treatment goals.

1 More than one options was possible. ^2^ Included the following treatment goals: abstinence required for access to somatic care; need for somatic health care; nutrition care; physical harm reduction.

Compared with patients with a drug use disorder, those with AUD were significantly more likely (*p* < 0.05) to report reduced substance use (as opposed to quit using substances) as their main treatment goal. Patients with a drug use disorder more frequently reported keeping custody of own children as an additional treatment goal compared with patients with primary AUD.

### 3.3 Multivariate comparisons between patients with AUD versus a drug use disorder

Several of the variables that differentiated between the two groups in the bivariate analysis remained significant in the multivariate logistic regression analysis. Having AUD was associated with older age, higher educational level, higher income from work, living in permanent housing, and having good relationships with friends. As displayed in Table 4, the significant odds ratios were highest for age group. In particular, patients with AUD were more likely to be in the age group >49 years than in the youngest age category (18–23 years). The observed differences in treatment goals between patients with AUD and a drug use disorder disappeared when age and sociodemographic variables were controlled for.

**Table 4:** Multiple logistic regression results. Odds ratios (ORs) and 95% confidence intervals (CIs) for characteristics of patients with alcohol use disorder (1) versus drug use disorder (0).

Variable	OR	95% CI		*p* value
*Age: Reference (18–23 years)*				
24–29 years	1.01	0.48	2.11	0.978
30–39 years	1.46	0.77	2.76	0.248
40–49 years	6.67	3.51	12.65	0.000
≥50 years	24.81	11.72	52.55	0.000

*Educational level: Base (High educational level)*				
Medium education	0.45	0.23	0.90	0.023
Low education	0.33	0.16	0.67	0.002

*Income: Reference (Income from labor)*				
Health-related benefits	0.51	0.27	0.95	0.034
Other economic support	0.33	0.16	0.70	0.004

*Marital status: Reference (Married/cohabitating/partnered)*				
Separated/divorced/widow/widowed	1.44	0.78	2.65	0.250
Single/unmarried	1.48	0.87	2.51	0.149

*Housing situation: Reference (Owned home)*				
Rented housing in private market	0.98	0.59	1.62	0.924
Rented municipal housing	0.55	0.31	0.99	0.045
Without permanent residence	0.45	0.25	0.79	0.006

*Relationships with friends: Reference (Very good)*				
Good	1.03	0.60	1.77	0.916
Poor	0.79	0.44	1.44	0.444
Very Poor	0.52	0.27	0.99	0.047

*Treatment goal*				
Quit using substances	0.72	0.44	1.18	0.191
Keep custody of children	0.72	0.40	1.30	0.276
Constant	1.14	0.36	3.67	0.824

Note. N = 931.

### 3.4 Robustness testing

Possible multicollinearity among the variables included in the regression model can reduce the statistical significance of the independent variables. We tested the robustness of the results by excluding and including different independent variables in separate regression analyses. For instance, removing marital status and housing situation did not change the results, nor did excluding/including either or both the source of income and educational level. In addition, including both, versus only one, of the social relationship variables (relationship with family and relationships with friends) in separate regression analyses did not alter the results.

The higher proportion who reported keeping custody for children as a treatment goal among those with drug use disorders could be attributed to the fact that they were younger than the patients with AUD, thus more likely to have responsibility for children (i.e. the treatment goal was relevant). To test for this association, we analyzed the difference between the groups in probability of reporting this treatment goal, adjusting for age. The results of this additional analysis revealed that the child custody treatment goal difference between groups changed to non-significant when age was controlled for (OR = 0.69, CI: 0.402–1.196, p = 0.188).

## 4 Discussion

Consistent with our hypothesis, patients with AUD reflected a less marginalized profile compared with patients with drug use disorders. The differences in social and sociodemographic characteristics accounted for the observed differences in treatment goals between the two patient groups.

The results showed that AUD was the most prevalent substance-specific disorder which reflects that alcohol is generally more widely used in the general population than illegal substances (Falk, Yi, & Hiller-Sturmhöfel, 2008). The observed age difference between patients with AUD versus drug use disorders are similar to those previously reported in analyses of patients admitted for SUD treatment and show that older patients are more likely to present with AUD (Lubman et al., 2016, Schulte and Hser, 2013). In contrast to people who abuse alcohol (Vaillant, 2003), those who become addicted to illicit drugs may rapidly develop a criminal lifestyle and involvement with the criminal justice system (Hser et al., 2001, Lubman et al., 2016). Consequently, given their drug use behaviors, people with a primary drug use disorder may experience stronger external pressure for SUD treatment and a perceived need for professional treatment at an earlier age compared with those with primary AUD.

Among the sociodemographic variables included in the current analyses, educational level and the source of income were identified as uniquely differentiating between patients with AUD versus those with drug use disorder. For example, almost 20% of patients with AUD had a higher educational level versus only 3% of those with a drug use disorder. Our findings are consistent with those of previous research comparing patients with AUD versus drug use disorders in SUD treatment (Lubman et al., 2016). The current findings add to the existing literature by showing that educational level, source of income, and housing situation contribute independently to differentiate patients with AUD from those with a drug use disorder. These results suggest that patients with AUD have a higher degree of “physical capital” than do patients with drug disorders (Best & Laudet, 2010). This knowledge may be relevant to research on the SUD population and for clinicians in their work facilitating recovery from SUD.

Both patients with AUD and those with a drug use disorder rated their relationship with friends as lower in quality than their relationship with family. However, we found significant differences between the two patient groups in their rating of social support from friends. Consistent with previous research (Stenius et al., 2010), patients with a drug use disorder more frequently reported poor relationships with friends compared with patients with AUD. One possible explanation may relate to the differences in the type of social networks between the patient groups. Use of illegal drugs often involves joining in social networks with other people using drugs, who might exert a negative influence on substance use behaviors (Dobkin, Civita, Paraherakis, & Gill, 2002), including criminal activity (Hser et al., 2001, Lubman et al., 2016). With the recognition of a serious drug use problem and the need for SUD treatment, it may become necessary to distance oneself from people using drugs (Bathish et al., 2017, Best et al., 2012). Thus, for many of those with drug use disorders entry into SUD treatment could mean losing friends. On the other hand, people with AUD may be more likely to belong to non-abusing social networks (Stenius et al., 2010) whose members support abstinence (Witkiewitz et al., 2017), and friendship persists throughout the treatment process.

The rating of family relationships did not differ significantly between patients with primary AUD and those with a drug use disorder. In both groups, about 80% of patients reported good family relationships. This finding is consistent with previous research suggesting that support from family may facilitate for entry into SUD treatment (Hser, Maglione, Polinsky, & Anglin, 1998). Drug treatment in Norway emphasizes the involvement of the patient’s family to help to understand the patient’s problems and to provide the best possible treatment and services (Norwegian Directorate of Health, 2016). The present finding suggests the importance of involving relatives in treatment for most SUD patients.

The expected difference between persons with AUD versus drug use disorders in report of reduced substance use as opposed to stop using substances as a treatment goal (Rosenberg & Davis, 2014) was found in bivariate analysis, but not after we controlled for social and sociodemographic factors in the multivariate model. The result may suggest that although reduced use may be a more acceptable treatment goal for persons with AUD (Rosenberg & Davis, 2014), a person’s total life situation, for example social and sociodemographic conditions, is considered when the treatment outcome goal is specified. Other factors that were not included in the current study, such as the severity of substance use problems (Rosenberg and Davis, 2014, Rosenberg and Melville, 2005) might also have been considered when the treatment goal was set.

The higher proportion of patients who reported to keep custody of children as a treatment goal among persons with drug use disorders compared with those with AUD was mostly explained by the age differences between the groups. Because persons with drug use disorders were younger, they were more likely to have young children. Parental AUD or drug use disorder is associated with a variety of negative outcomes for the children (Marmorstein, Iacono, & McGue, 2009), and having concerns about custody for children may be a motivating factor for entering SUD treatment (Swift, Copeland, & Hall, 1996). The present results may suggest that persons with AUD and drug use disorders who received inpatient SUD treatment equally emphasized responsibility for their children, irrespective of their living situation.

### 4.1 Strengths and limitations

The strengths of this study include the relatively large sample size and a high response rate, which makes major selection biases unlikely and enables generalizability of the findings to the inpatient SUD treatment population in Norway.

However, the study has several limitations that should be considered. In the current analyses, patients were grouped according to their main substance use disorder for the index treatment stay, but the data did not include information about any additional substance use problems. Therefore, within both groups, there probably were individuals with combined alcohol and drug use problems. It is possible that restricting the group of patients with primary AUD to those who exclusively used alcohol, may have allowed the identification of other characteristics that distinguished patients with primary AUD from those with a drug use disorder.

We did not have any information about the patients who were not included in the census. Because the study had a cross-sectional design and included patients receiving inpatient treatment on a single day, those undertaking longer treatment (i.e., with more severe substance use problems) were more likely to have been included. However, there is no reason to believe that the study had bias toward the inclusion of patients with AUD versus other drug use disorders.

Data were collected in November 2012. Although it is unlikely that typical characteristics of patients with AUD versus drug use disorders have changed over the years, the attitudes to the use of drugs may have done so. For example, the perceived risk associated with cannabis use has decreased in recent years (Carliner et al., 2017, Hasin, 2018). It is therefore possible that a more recent study on these issues would have shown reduced drug use (as opposed to abstinence) to be more widespread as a treatment goal among the patients. Additional research is needed to investigate this further.

When conducting the study, which was an assignment from the Norwegian Directorate of Health, we emphasized achieving a high response rate while at the same time covering a wide range of topics. Consequently, the data collection did not include in-depth interviews with patients. Our results are therefore limited as concerns the roles of social relationships and treatment goals, and the factors that may influence these variables. Due to the cross-sectional design, no causal inferences can be drawn from the present study.

Limitations also include the use of a non-validated scale to assess the patients’ ratings of their relationships with friends and family. Further research should examine differences in social relationships between patients with AUD versus drug use disorders using standardized and more detailed instruments. Finally, the current study was limited to comparing persons treated for AUD with persons treated for all types of illicit drug use disorders. Future research may want to study the differences in characteristics between groups of patient with different drug use disorders.

## 5 Conclusions

Patients with primary AUD and a drug use disorder differed on typical sociodemographic factors (e.g., age, education, employment, housing situation), and these factors explained differences in treatment goals between the groups. The current findings may inform strategies for prevention, treatment and follow-up, and be of relevance when conducting research on the SUD treatment population. The lower frequency of perceived support from friends among patients with a drug use disorder suggests a need for targeted efforts to help reestablish or build supportive, nonabusive relationships with friends.

## Role of Funding Sources

The Norwegian Directorate of Health funded the data collection. The funding source had no role in the study design, data collection, analyses, writing, or the decision to submit the manuscript for publication.

## CRediT authorship contribution statement

**Helle Wessel Andersson:** Conceptualization, Writing - original draft. **Solfrid E. Lilleeng:** Data curation, Supervision, Validation. Solveig **Osborg Ose:** Conceptualization, Formal analysis, Methodology, Supervision, Validation.

## Declaration of Competing Interest

The authors declare that they have no conflicts of interest.
